# The Middlesex Hospital

**Published:** 1904-05-07

**Authors:** 


### THE MIDDLESEX HOSPITAL.

### THE CANCER RESEARCH DEPARTMENT.

The proceeds of the festival dinner of the Middlesex Hospital, which was held at the Whiteball Rooms, on Tuesday, are to be devoted to the purposes of (1) making good the inroads on invested capital which have been necessitated by new buildings and other improvements to meet the demands of modern requirements; (2) for the maintenance of the Cancer Charity, which was established in a small ward for 10 or 12 patients, in 1792, and which now occupies a wing of the hospital to itself, with accommodation for 45 patients, and which treated 156 in- and 173 out-patients in 1903; (3) for the maintenance of the special department for research into the nature and cure of cancer, established in 1900; (4) for keeping up the Hospital's Convalescent Home at Clacton; and (5) to provide for the improved and extended accommodation for the nurses. In all, a sum of £6,000 was collected as the result of the appeal.

After the usual loyal toasts,

The Lord Mayor, who occupied the chair, proposed "The Middlesex Hospital," which was founded, he said, over 150 years ago. One hundred and ten years ago the hospital introduced a feature which had made it memorable amongst the hospitals of the City, viz., a cancer ward for the treatment of a disease which he was told was increasing with great rapidity.

### A Ten Years' Review.

Lord Sandhurst, in responding, said he was not going to inflict upon his hearers a number of statistics. Those were to be found in the annual report and in the appeals of the hospital. Instead, he would like to give them a short review of what the hospital had done during the ten years which had elapsed since the King (then the Prince of Wales) honoured the hospital by presiding at its festival dinner. (Applause.) Since 1894, the year in which this took place, the new cancer wing had been opened, a wing which, it was not too much to say, was a model of what a hospital should be in construction and in equipment. It had its own matron and its own staff of nurses and servants, and it had enabled the hospital to set aside that portion formerly occupied by cancer cases for other purposes. The convalescent home and branch hospital had been opened, and that terrible eyesore in the basement of the Middlesex Hospital, the laundry, had been done away with, and a site acquired at Hendon where a commodious laundry had been built. They had also moved the kitchen from the basement to the top of one of the wings, with the result that they had been able to arrange in the basement for a cold storage, a disinfecting chamber, and for greatly increased steam power. The hospital, too, had added a clinical laboratory, and a laboratory for cancer research—(hear, hear)—and a building for Finsen light and *x*-ray treatment, from which great benefit would come and where very useful work would be done. Then, by no means last, they had been able to build, owing to the falling in to them of some houses in Cleveland Street, a commodious and comfortable home for the nurses, which, in addition to providing for the comfort of the staff, had enabled them to greatly augment the number of nurses, thereby lessening their hours of work. (Applause.)

### Cancer Investigation.

The hospital was endeavouring to do a great deal in the way of cancer research. The second report of the Cancer Research Department, covering some 230 pages, had just been issued, and there were many matters of great interest in it, and especially so was one paper on "Heredity in Cancer." During the last six months, too, the hospital records since 1746 had been investigated, with the result that the cancer department was now ready to deal with material ranging over some 6,000 cases, a collection of unusual value and public utility. (Applause.) There was one thing which rather hampered their cancer investigations, and that was that they were in a difficulty about funds, for this reason, that they could not, unless the money was subscribed *ad hoc*, give away the funds of the hospital in large salaries, and they required for that class of work the best brain power that the world could produce. (Hear, hear.)

Acknowledgments.

Lord Sandhurst thanked Lord Derby for his generosity in giving a donation of £1,000 towards the hospital—(loud applause)—and also Lady Derby for taking upon herself the chairmanship of the committee of ladies whose efforts were of the greatest, and whose results would be, he was sure, in proportion to those efforts. (Applause.) In conclusion his lordship paid a warm tribute to the work done by the permanent officials, Mr. Clare Melhado (the secretary-superintendent), Mr. Fardon (the resident medical officer), Miss Thorold (the lady superintendent), and the Rev. H. E. Gunson (the chaplain). (Applause.)

London's Lack of Esprit de Corps.

Lord Sandhurst proposed "The Medical Staff," which was responded to by Mr. Henry Morris and Dr. J. Kingston Fowler. The latter said that all was not well with the medical schools of London. They were suffering from the competition of the Universities which were springing up, he was glad to see, all over the country. In consequence of that the medical schools of London were suffering from a paucity of endowments for laboratories and other things which were necessary for the teaching of scientific medicine. He observed that the first member of the new Education Committee of the London County Council was a medical man. (Hear, hear.) He hoped they might look to Sir William Collins to convert that body to the view that medical education was a branch of technical education in which the general public was as interested as in any of the others—(hear, hear)—and that some of what was called the "whisky money" might be devoted to medical education. (Laughter.) The people of this country had yet to learn the lesson which had been learnt in America, Germany, and Japan, that the best investment of the national funds was the endowment of the highest forms of education and research. (Applause.)

The health of "The Chairman" concluded the toast list.

